# Predicting Residence Time and Melt Temperature in Pharmaceutical Hot Melt Extrusion

**DOI:** 10.3390/pharmaceutics15051417

**Published:** 2023-05-06

**Authors:** Judith Winck, Tobias Gottschalk, Markus Thommes

**Affiliations:** 1 Laboratory of Solids Process Engineering, Department of Biochemical and Chemical Engineering, TU Dortmund University, Emil-Figge-Str. 68, 44227 Dortmund, Germany; judith.winck@tu-dortmund.de (J.W.); tobias.gottschalk@tu-dortmund.de (T.G.); 2 Drug Delivery Innovation Center, INVITE GmbH, Chempark Building W32, 51368 Leverkusen, Germany

**Keywords:** hot melt extrusion, solid dispersion, process parameter, quality by design, one dimensional, simulation, model

## Abstract

Hot-melt extrusion is increasingly applied in the pharmaceutical area as a continuous processing technology, used to design custom products by co-processing drugs together with functional excipients. In this context, the residence time and processing temperature during extrusion are critical process parameters for ensuring the highest product qualities, particularly of thermosensitive materials. Within this study, a novel strategy is proposed to predict the residence time distribution and melt temperature during pharmaceutical hot-melt extrusion processes based on experimental data. To do this, an autogenic extrusion mode without external heating and cooling was applied to process three polymers (Plasdone S-630, Soluplus and Eudragit EPO) at different specific feed loads, which were set by the screw speed and the throughput. The residence time distributions were modeled based on a two-compartment approach that couples the behavior of a pipe and a stirred tank. The throughput showed a substantial effect on the residence time, whereas the influence of the screw speed was minor. On the other hand, the melt temperatures during extrusion were mainly affected by the screw speed compared to the influence of the throughput. Finally, the compilation of model parameters for the residence time and the melt temperature within design spaces serve as the basis for an optimized prediction of pharmaceutical hot-melt extrusion processes.

## 1. Introduction

Hot melt extrusion was established as a common unit operation in pharmaceutical applications in the early 1970s [1]. Today hot melt extrusion is applied to produce drug products with additional value such as drug-loaded medical devices, sustained-release dosage forms and highly bioavailable drug formulations [2,3]. In the majority of the latter applications, a crystalline drug substance is dissolved in a polymeric carrier during the manufacturing procedure and a molecularly dispersed mixture called an amorphous solid dispersion (ASD) is obtained [4]. Therefore, the polymeric excipient is usually processed above its glass transition temperature so that a viscous liquid is obtained in which the drug substance is subsequently dissolved [5,6]. The high mechanical stress in the extrusion process favors dispersive and distributive mixing and thus supports the dissolution process, overcoming limitations associated with the high viscosity. Nevertheless, the dissolution process is constrained by the processing time and temperature, since the solubility of the drug within the polymer melt will be a function of temperature, which defines the dissolution rate [7,8].

Co-rotating twin screw extrusion is the most common technique in HME because of its favorable mixing capacity. Here, two intermeshing screws transport the material from a feeding port toward a die within an extrusion barrel. This continuous process is affected by numerous critical material attributes (e.g., powder density and melt rheology) as well as critical process parameters (e.g., throughput and screw speed) [9]. The interconnection of these influencing variables makes the process design quite challenging since relevant dependent critical process parameters (e.g., melt temperature and the residence time) cannot be set directly. Therefore, deeper knowledge about the interconnection of the process influencing variables is required in order to successfully design a hot melt extrusion process and thereby achieve the desired product specification.

There are numerous studies investigating the residence time of various materials in pharmaceutical hot melt extrusion [10,11]. Generally, governing equations contain a lag term, which is mainly related to the axial transport of the material through the extruder, as well as a mixing term that accounts for mixing in the axial direction [12]. Based on this, each molecule experiences a certain probability of remaining for a shorter or longer time within the process. This is represented by a probability density function or residence time distribution function (E(t)). However, the residence time distribution can be seen as an overall process time distribution which includes feeding, transport, mixing and dissolution. Therefore, the residence time is not equal to the dissolution time of the drug in the polymer mentioned earlier. Moreover, an axial temperature gradient causes a varying solubility of the drug in the polymer.

The material or melt temperature is one key variable of the hot melt extrusion process since it determines the melt viscosity during processing as well as the solid-state properties of the final product [13]. When preparing ASDs via hot melt extrusion, high temperatures enhance the drug dissolution within the polymer, whereas particularly high temperatures lead to thermal degradation and potentially toxic impurities [14]. The melt temperature can be adjusted via heat conduction through the extruder barrel but is also related to material processing (e.g., melting, water evaporation). The conversion from mechanical (shearing by the screw) to thermal energy has a significant impact on the melt temperature. In production-scale extrusion processes, all the energy is usually applied by the extruder screw so that no external barrel heating is needed to plasticize the materials. The systematic design of extrusion processes that are based solely on the application of mechanical energy is called autogenic extrusion and leads to a more robust and scalable process. Therefore, autogenic extrusion without barrel heating was the focus of this study.

In a previous investigation [15] the throughput and the melt temperature were studied in autogenic extrusion, running the extruder at the capacity limit (constant specific feed load) at various screw speeds. Thereby, the bulk density of the powder material was found to be the relevant parameter with respect to the maximum throughput. The melt temperature was mainly related to the polymer rheology, and it increased with throughput and screw speed. A model was developed to describe this behavior quantitatively. However, it was not possible to alter the melt temperature independently of the throughput. This can be seen as a limitation due to the particular importance of product properties.

The aim of this study was to extend the theoretical framework from previous investigations, finding a mathematical model to describe the influence of throughput and screw speed on residence time and melt temperature. This novel approach of coupling the prediction of residence time and melt temperature in autogenic extrusion is highly relevant in pharmaceutical hot melt extrusion since these two process parameters are key factors for assessing drug dissolution in the polymer. Thus, it is possible to predict and propose optimized process conditions that enable the preparation of ASDs, while minimizing energy consumption and drug degradation.

## 2. Materials and Methods

### 2.1. Materials

Three polymers commonly used in pharmaceutical hot melt extrusion were utilized in this study as model materials. Poly-vinylpyrrolidone-vinylacetate (PVPVA) (Plasdone S-630, Ashland Inc., Columbus, OH, USA), graft co-polymer Soluplus (SOL) (BASF SE, Ludwigshafen, Germany) and basic butylated methacrylate copolymer (bBMA) (Eudragit EPO, Evonik Industries AG, Darmstadt, Germany) were selected (rheology parameters are given in Table 1).

### 2.2. Hot Melt Extrusion

**Figure 1:**
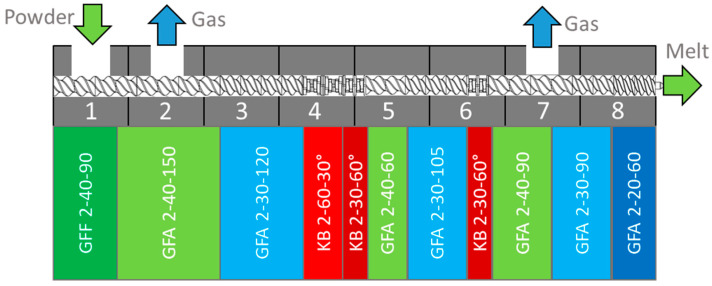
Screw and barrel configuration [15]. Green and blue symbolize conveying elements, red marks kneading zones. Nomenclature according to Leistritz.

A loss-in-weight feeder (K-Tron K-ML-SFS-KT20, Coperion, Niederlenz, Switzerland) was used for dosing the powder material in the extruder. The extrusion experiments were carried out in a co-rotating twin screw extruder (ZSE 27 MAXX, Leistritz, Nuremberg, Germany) containing modular screw elements with a 28.3 mm diameter and a length of 32 D (Figure 1). A heated extrusion die with a 3 mm diameter and 11.7 mm length was utilized, and the die pressure was measured by a pressure transducer (KE1-7-M-B35D-1-4-D-S-P-E, Gefran, Provagilo d’Iseo, Italy).

The screw and barrel design were similar to previous investigations [16]. For all operating conditions, the melt temperature at the die was measured in triplicate with an IR-camera (TESTO 875, Testo SE & Co. KGaA, Lenzkirch, Germany) using the material-specific emission coefficients (PVPVA, SOL, bBMA: 0.93, 0.96, 0.93) when the torque and the pressure at the die had reached a constant value (equilibrated state). For the autogenic extrusion, the measurement was conducted after the barrel reached a constant temperature.

### 2.3. Residence Time Determination

For the residence time determination, the marker substance quinine-dihydrochloride (Caesar & Loretz, Hilden, Germany) was utilized with a fraction of 16–23 mg per polymer mass flow of 1 kg/h. The marker was added as a Dirac-impulse through the hopper of the extruder, and the response signal was measured in the die with an inline UV-Vis-spectrophotometer (Inspectro X, ColVisTec AG, Berlin, Germany) at 250 to 650 nm. This was performed in transmission with two probes (TPMP, ColVisTec AG, Germany) at an offset of 180°. The transmission, expressed as the ratio of transmitted light intensity to impinging light intensity, is converted to absorbance using the Lambert-Beer law.

### 2.4. Rheological Properties

The rheological properties of the polymers were taken from the literature (Table 1). To do this, a model was used, coupling the approaches from Carreau and Arrhenius [17].

(1) $$\eta =\frac{\eta_{0}a_{T}}{{\left(1+\frac{\dot{\gamma }a_{T}}{{\dot{\gamma }}_{c}}\right)}^{c}}$$

(2) $$a_{T}=e^{\frac{E_{A}}{R}\left(\frac{1}{T}-\frac{1}{T_{Ref}}\right)}$$

In accordance with Carreau (Equation (1)), the dynamic viscosity (η) is a function of the shear rate ($\dot{\gamma })$ and three material-dependent parameters (zero-shear viscosity ($\eta_{0}$), critical shear rate (${\dot{\gamma }}_{c}$) and flow exponent (c)). The shift factor ($a_{T}$) accounts for the temperature (T) dependency of the viscosity and is given by the Arrhenius equation (Equation (2)), where a material-dependent activation energy ($E_{A}$) and reference temperature ($T_{Ref}$) are used.

**Table 1:** Rheology parameters for the polymers based on a refit of literature data using the Carreau-Arrhenius approach.

Substance	$\eta_{0\;}$ [Pa s]	${\dot{\gamma }}_{c}$ [1/s]	c [−]	$E_{a}$ [kJ/mol]	$T_{Ref}$ [K]
PVPVA [18]	169.7	133.1	0.387	198.3	473
SOL [19]	147.3	136.7	0.411	150.8	473
bBMA [19]	25.58	1688	0.561	140.3	473

## 3. Results and Discussion

### 3.1. Experimental Design

In a previous investigation, a systematic study was performed to evaluate the melt temperature as a function of throughput using the capacity limit of the extruder. At this limit, the maximum specific feed load is reached, which sets the volume flow of the material (Equation (3), numerator) in relation to the theoretical transport capacity of the extruder (Equation (3), denominator). Thus, the specific feed load (SFL) is a function of the mass flow ($\dot{m}$), the material density ($\rho_{material}$, 1190 kg/m^3^ for PVPVA, 1080 kg/m^3^ for SOL and 1092 kg/m^3^ for bBMA [15]), the screw speed (n) and the screw diameter (d).

(3) $$SFL=\frac{\dot{m}/\rho_{material}}{n\cdot d^{3}}$$

Different maximum SFL values were found for the three considered polymers, which was attributed to the differences in bulk density. The lowest value (SFL=0.0309) found for PVPVA was intended to be used as the highest value for all three polymers in this study, to guarantee that all desired SFL values are accessible. However, deviating SFL values were chosen for bBMA due to differences in the material density. The highest mass flow rate was set to 12 kg/h, which was in the center of the operating range for all three polymers in the previous study. Fractions of 12 kg/h, namely 6 kg/h and 3 kg/h, were used as lower factor levels in terms of a design of experiments. The screw speed was adjusted to obtain the desired SFL value (SFL=0.0309), which was different for the polymers based on differences in material density. The screw speed was doubled and quadrupled to generate additional factor levels for the screw speed at lower SFL values (Appendix A Table A1). Two repetitions were performed at the center point of the design space to assess the experimental error in terms of a design of experiments.

**Figure 2:**
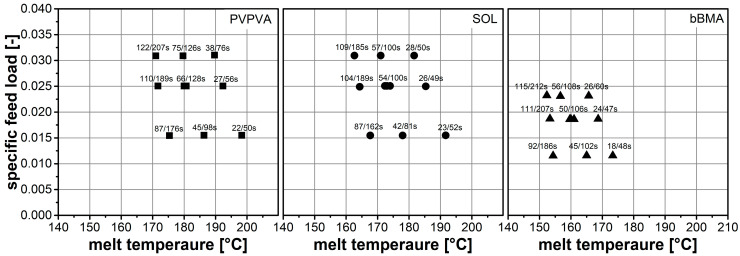
Results of extrusion experiments are given in SIOS plots. Mass flow rate and screw speed were varied systematically, leading to differences in specific feed load. The resulting measured melt temperature is given on the abscissa. The residence time is represented by the numbers giving 10% (t_10_) and 90% quantiles (t_90_) of the distribution.

The results of the extrusion experiments are visualized in an extrusion diagram known from the Scale Independent Optimization Scheme (SIOS) [20], where the specific feed load is plotted over the measured melt temperature at the die (Figure 2). Additionally, the 10% and 90% quantiles of the residence time distributions are given as numbers next to the data points.

Generally, higher SFL values lead to longer residence times, which is attributed to a higher barrel load resulting in a higher hold-up. Considering one specific SFL value, the horizontal alignment of the data points is related to the throughput increasing from left to right. Shorter residence times are found at the right since higher throughput through a confined volume leads to shorter residence times.

In terms of the melt temperature, lower values are found at higher SFL, since less shear is applied to the material at lower screw speeds, causing less energy dissipation. For one SFL, the horizontal alignment of the data points also includes a change in screw speed, increasing from the left to the right. The increase in temperature can be attributed to an increased shear rate, resulting in higher energy dissipation.

There are remarkable differences between the polymers in the melt temperature, which might be related to differing melt rheology as found previously [15]. In this respect, differences in residence times were minor.

### 3.2. Residence Time Determination

Pharmaceutical hot melt extrusion is usually a continuous process where multiple sub-processes are performed sequentially in a co-rotating twin screw extruder. Common residence-time models for hot melt extrusion distinguish between material conveying and mixing [12]. In this study, the two-compartment model of Reitz was used to express the experimental data [21], which couples the residence time behavior of a pipe and a stirred tank (Equation (4)).

(4) $$c\left(t\right)=\frac{1}{2}c_{0}exp\left(\frac{1}{2}k^{2}\sigma^{2}-k\left(t-t_{dead}\right)\right)\cdot erfc\left(\frac{k\sigma^{2}-\left(t-t_{dead}\right)}{\sigma \sqrt{2}}\right)=\frac{c_{0}}{k}E(t)$$

This model comprises three parameters that describe the residence time distribution. The dead time ($t_{dead}$) is related to the residence time of plug flow through a pipe. The standard deviation (σ) gives the corresponding back mixing, which is frequently interpreted as a backflow of material. The main mixing action is described by a rate constant (k) which can be thought of as a rate constant of a dilution process within a continuously stirred tank. Besides these model parameters, the time (t) and a scaling factor ($c_{0}$) are used to derive the residence time density function (E(t)).

(5) $$V_{dead}=t_{dead}\dot{V}$$

(6) $$V_{mix}=\dot{V}/k$$

(7) $$CV=\sigma /t_{dead}$$

The dead time and the rate constant are usually put in the perspective of the used volume flow ($\dot{V}$) leading to the dead volume ($V_{dead}$) and mixing volume ($V_{mix}$), respectively. The standard deviation is frequently normalized to the dead time and expressed as a coefficient of variation (CV) (Equations (5)–(7)). These transformations lead to more comparable parameters independent from the process conditions, namely the throughput (volume flow).

**Figure 3:**
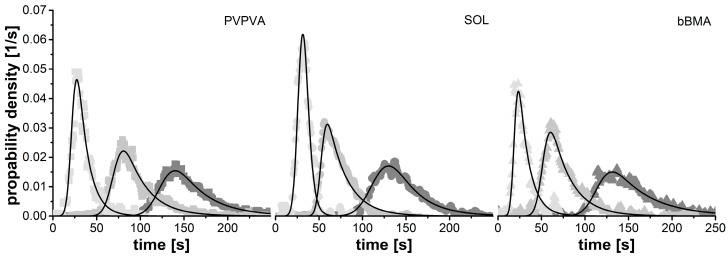
Representative residence time distribution measurements and the corresponding fitted models. The largest (light grey) and smallest (dark grey) residence time distributions are shown for each polymer as well as data from center points (medium grey).

The two-compartment model fits the experimental data quite well. In Figure 3, the residence time distributions of the extrusion experiment with the lowest, medium and highest residence time are given for each polymer. The corresponding model parameters for all 33 extrusion experiments are provided in Table A1. Overall, short residence times are related to narrow distributions while long residence times tend to be wider. This observation is quite in line with the literature [22,23,24]. When comparing the residence time distribution functions between the polymers, no remarkable differences were observed.

### 3.3. Residence Time Evaluation

A more detailed statistic evaluation of the residence time was performed using variance analysis. Thereby, specific feed load (SFL), melt temperature (T) and polymer type (PVPVA/SOL/bBMA) were used as influencing factors, while the parameters of the two-compartment model ($V_{dead},V_{mix},CV$) served as response variables (Equation (8)).

(8) $$response=\beta_{0}+\beta_{1}T+\beta_{2}SFL+\beta_{3}SOL+\beta_{4}bBMA$$

The individual model parameters ($\beta_{i}$) were fitted by multiple linear regression (Modde 10.1.0, Sartorius Data Analytics, Umea, Sweden) while insignificant terms were removed in order to strengthen the model (backward regression). The significant (α=0.05) model parameters, as well as the quality indicators such as coefficient of determination, coefficient of prediction, lack of fit and repeatability, (RP) [25], are given in Table 2.

The quality of all three regression models is rather low, as indicated by the low coefficient of determination and coefficient of prediction values, which ideally should be close to one. The repeatability is fairly high at the center point. However, it might be different at other points in the design space, which could cause the invalidity of the model. Looking at the raw data, the data points tend to scatter at experiments that were performed at low throughputs within the entire dataset.

The dead volume is only affected by the specific feed load, which is a measure for the use of the transport capacity of the extruder. The specific feed load also represents the barrel load. Therefore, higher SFL values lead to higher barrel loads (hold up), which results in a higher dead volume in the residence time model.

The mixing volume depends on the used polymer: where PVPVA is the standard in this regression model, SOL is lowering and bBMA is increasing the mixing volume. In initial attempts, this behavior was correlated with the rheological properties of the individual polymers. This was not successful. However, a correlation was found for the bulk density of the polymer (315 kg/m^3^ for PVPVA, 597 kg/m^3^ for SOL and 339 kg/m^3^ for bBMA [15]), where higher densities led to a lower mixing volume ($\beta_{\rho }=-4.8\pm 2.29)$. This observation is plausible since the powder is also mixed in the unmolten (dry) state in the extruder. Nevertheless, this effect was not considered in the subsequent model since the influence of density on the mixing volume was low and the data basis of this observation was rather small (PVPVA and bBMA have similar bulk densities).

The most interesting response with respect to the statistical evaluation is the coefficient of variation of the residence time distribution because it is influenced by all three factors. Even if this effect is statistically significant, it is, practically speaking, not relevant, since the coefficient of variation is nearly constant, and the width of the residence time distribution is dominated by the rate constant (k). Therefore, these effects were ignored in further investigations.

### 3.4. Residence Time Prediction

Utilizing the findings of the two previous paragraphs, the aim was to predict the residence time distribution of the hot melt extrusion process. Therefore, the two-compartment model of Reitz was combined with the statistic evaluation of the experimental data using a constant mixing volume ($V_{mix}=30.8\;mL$) and constant coefficient of variation (CV=0.134) but linear function ($\beta_{0}=44.5\;mL,\beta_{2}=1667\;mL$) for the dead volume ($V_{dead},$ Equation (9)).

(9) $$V_{dead}=\beta_{0}+\beta_{2}SFL$$

**Figure 4:**
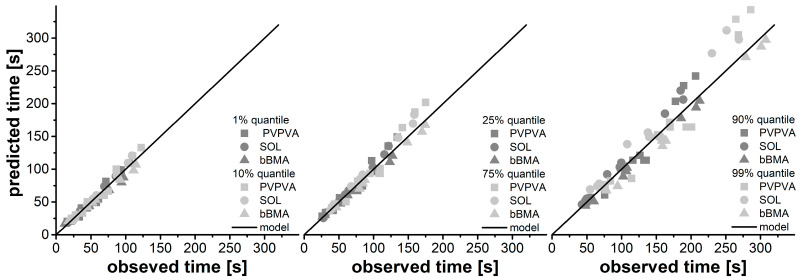
Correlation between observed and predicted quantiles of the residence time distribution used as a performance indicator of the predictive power of the residence time model.

Using those four model parameters, residence time distributions were calculated for all 33 performed experiments. These predicted data were compared to the measured data using the quantiles of the residence time distribution function (Figure 4).

The predictions fit quite well with the experimental observations. There are no systematic deviations between the modeled and the experimental data, but the deviations seem to increase at higher quantiles. This observation was studied further by calculating the coefficient of variation which increases from 8% to 10% when changing from the 10% quantile to the 90% quantile. This was attributed to the error propagation for uncertainties in the model parameters within the distribution function since the errors tend to accumulate with respect to time in these cumulative functions. The overall deviation might be reduced by using more complex prediction models including the bulk density of the material. However, the coefficient of determination of repetitive measurements in the experimental data was 6%. Finally, 8% to 10% of the model predictions were considered to be reasonably good.

### 3.5. Melt Temperature Prediction

In a previous study [15] the melt temperature in hot melt extrusion was predicted at the capacity limit of the extruder when running at a constant specific feed load and keeping throughput and screw speed in a fixed ratio. Within this study, the throughput and screw speed were varied independently from each other, and the influence on melt temperature was investigated. Therefore, the rheological behavior of the polymers was required and implemented by a coupling of approaches of Carreau and Arrhenius (Equations (1) and (2)).

The melt viscosity at the die ($\eta_{die}$) was calculated with the Hagen–Poiseuille Law [26], using the correction of Weissenberg–Rabinowitsch [27] in its simplified form valid for fluids that follow the Ostwald–de Waele power-law model, in order to account for the shear-thinning behavior of the polymer melts.

(10) $$\eta_{die}=\frac{{∆p}_{die}r_{die}}{2l_{die}}\cdot \frac{\pi r_{die}^{3}}{{4\dot{V}}_{die}}\cdot \frac{4-4c}{4-3c}$$

Therefore, the pressure drop at the die (${∆p}_{die}$), the geometry of a cylindrical die ($r_{die}$, $l_{die}$), the volume flow (${\dot{V}}_{die}$) and the flow index (c) were considered. In hot melt extrusion, the melt viscosity at the die is a hyperbola with respect to the screw speed (n) where a setup-specific correlation factor ($\tau_{extruder}$) is implemented as extruder-specific shear stress (Equation (11)).

(11) $$\eta_{die}=\tau_{extruder}\cdot \frac{1}{n}$$

**Figure 5:**
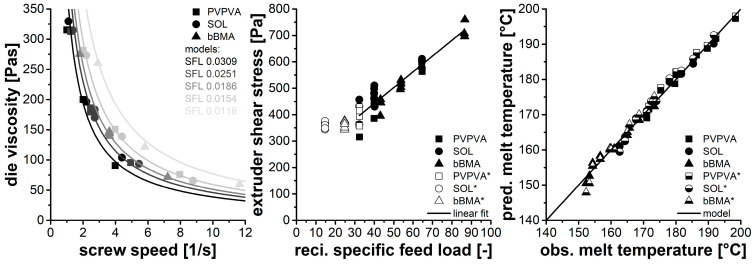
Die viscosity as function of screw speed modeled using specific feed-load dependent extruder shear stress (**left**), extruder shear stress related to reciprocal specific feed load—open symbols are data from a previous study * [15] (**middle**), correlation of measured and predicted temperature using model parameters from this study (closed symbols) and previous study * (half open symbols) (**right**).

In previous investigations, the extruder-specific shear stress was constant for different operating conditions, performing the extrusion experiments at the capacity limit [15]. However, in this study, a lower specific feed load led to differences in the extruder-specific shear stress (Figure 5, left). In order to elucidate this effect further, different extruder shear stresses were fitted to the experimental data, and a correlation to the specific feed load was found. Thereby, a lower specific feed load was related to higher extruder shear stress.

Based on this observation, the extruder shear stress was plotted as a function of the reciprocal specific feed load, and a linear correlation with slope ($s_{shear\;stress}$) and intercept ($i_{shear\;stress}$) was identified (Figure 5, mid, Equation (12)). The corresponding parameters are given in Table 3, while no relevant differences were found between the individual polymers. The coefficient of determination is quite high, which proves the correlation even if there is some scatter of the individual data points (Figure 5, mid).

(12) $$\tau_{extruder}=s_{shear\;stress}\frac{1}{SFL}+i_{shear\;stress}$$

Moreover, the data points of the previous study were visualized as well, serving for comparison (open symbols). There, the extruder shear stress was independent of the specific feed load looking at different polymers. This behavior was explained by differences in the polymer rheology and an inherent adjustment of the melt temperature to a corresponding melt viscosity at the die. In this study, the extruder shear stress is not constant but the same dependency to specific feed load was found for all three polymers. This contradiction between the studies can be solved by the higher SFL values used in the previous investigation. Apparently, there is a change in the extrusion regime at about SFL = 0.035 (1/SFL = 29), which might be indicated by the high scattering of the PVPVA data in this region as well.

Lowering the specific feed load below a critical value by lowering the throughput leads to less volume flow as well as less shear in the die and thus to a higher melt viscosity in the die. According to Equation (11), the viscosity at the die is related to the screw speed rather than the shear rate so the shear stress of the extruder has to increase in order to accommodate the increased viscosity. Assuming a fixed processing volume in the extruder, lower feed rates lead to less filling of the barrel, resulting in a hyperbolic influence of the specific feed load to the applied shear stress. When exceeding a certain value of the specific feed load, the processing volume is completely filled resulting in a constant extruder shear stress.

Based on these observations, the throughput and the screw speed can be transferred into a melt viscosity at the die using two material-independent parameters ($s_{shear\;stress}$, $i_{shear\;stress}$, Equation (11)). These can subsequently be used to predict the melt temperature, considering the polymer rheology (Table 1, Equations (1) and (2)) as well as two correction factors ($s_{extruder}$, $i_{extruder}$, Equation (13)), accounting for the non-ideal behavior of the extruder.

(13) $$log\frac{\eta_{die}}{\eta_{calculated}}=s_{extruder}\cdot log\frac{n}{n_{max}}+i_{extruder}$$

This procedure has been previously described [15], and the model parameters of the previous study were used to predict the melt temperatures found in this work (Figure 5 right, half open symbols). Additionally, the model parameters were also determined to form the actual data set (Table 3) and are visualized as well (Figure 5 right, closed symbols). No relevant difference between the two parameter sets was found, and the predictive power to estimate the melt temperature was considered to be high.

### 3.6. Design Space

**Figure 6:**
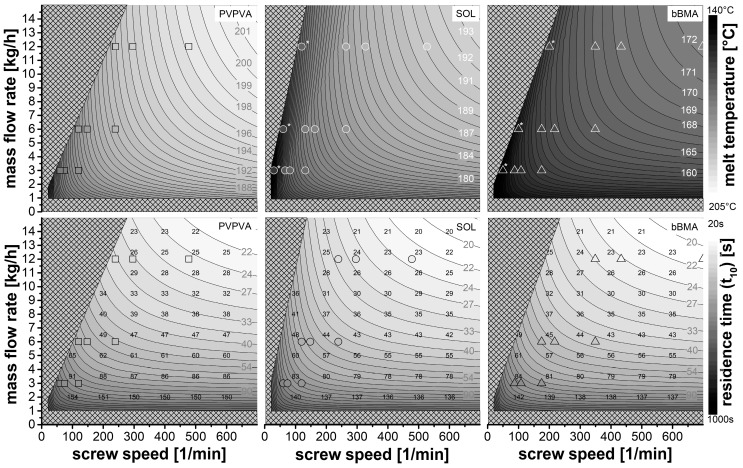
Melt temperature (**top**) and residence time (**bottom**) as a function of mass flow rate and screw speed for three polymers: the crosshatched area is not accessible for extrusion, the grayscale is melt temperature or the 10% quantile of the residence time distribution based on the models, symbols represent the experimental data in the grayscale of the contour plot, the black numbers indicate the span of the residence time distribution (t_90_-t_10_) in seconds, the data for the marked symbols (*) are taken from a previous dataset [15].

In the previous paragraphs, mathematical models were developed to predict melt temperature and residence time in pharmaceutical hot melt extrusion using experimental data. However, these models are complex and cannot be used intuitively. Therefore, these results were visualized based on common diagrams with throughput and screw speed on two axes. Individual diagrams are shown for the melt temperature (Figure 6, upper row) and the residence time (Figure 6, lower row) as well as for the polymers (Figure 6, columns).

The cross-hatched area is inaccessible for extrusion processes. Close to the abscissa at the throughput of less than 1 kg/h, there was a squeaking noise for all polymers, which indicated a contract between screw and barrel resulting in undesired wear. Below 20 rpm screw speed, the extrusion process is inaccessible due to the high torque of the extruder at low screw speeds. The crosshatched area close to the ordinate is material-dependent (bulk density) and related to the maximum specific feed load. At these feed rates, the screw speed is too low to transport the material through the extruder. Moreover, there is a material-dependent upper limit for the throughput which was found to be 30 kg/h for PVPVA, 42 kg/h for SOL and 36 kg/h for PVPVA, related to the capacity of the powder feeder and the bulk properties of the polymer.

The grayscale in those diagrams encodes the melt temperature and residence time, which were taken from the models discussed before. The data points of the extrusion experiments are also given in the diagrams and coded in the same grayscale used for the model. The marked symbols (*) were taken from a previous study [15] and served as reference points (melt temperature data only). The diagrams for the residence time refer to the 10% quantile of the residence time distribution. Additionally, the span (t_90_-t_10_) in seconds is provided as black numbers to describe the width of the residence time distribution.

The melt temperatures differ between the polymers. The highest temperatures were seen for PVPVA and the lowest for bBMA. This is related to the individual melt rheology of the polymers. However, the temperature distribution within the design space is quite similar. Higher throughput and higher screw speed lead to higher melt temperatures. The screw speed increases the shear rate in the extruder, leading to higher energy dissipation and higher melt temperatures. The throughput, in turn, increases the hold-up ($V_{dead}$) and the exposure to mechanical stress, which also increases the melt temperature. However, within the design space, the screw speed has much more influence on the melt temperature as compared to the throughput.

With respect to the residence time, the throughput dominated the process while the effect of screw speed was minor. Note that the grayscale uses a logarithmic transformation to cover the entire range. The width of the residence time distribution is correlated with the residence time itself since the span (t_90_-t_10_) and the 10% quantile led to similar values. Therefore, it is impossible to achieve high residence times and narrow size distributions as often desired just by varying throughput and screw speed. Overall, the residence times are rather short for dissolving a crystalline drug substance within the polymer melt [28]. Therefore, the process has to be designed for far above the solubility temperature taken from the phase diagram in order to avoid kinetic constraints.

In the literature, process design is frequently performed at low throughput, sometimes at low screw speed (lower left-hand corner). These process conditions with respect to melt temperature and residence time deviate highly from the production scale. That is why different product properties can be expected.

## 4. Conclusions

Pharmaceutical hot melt extrusion is an emerging but challenging manufacturing process due to its high number of process variables. In terms of amorphous solid dispersions, the material temperature and process time are considered to be critical process parameters, since the drug substance has to be dissolved in the polymer melt during manufacturing. In this work, these two critical process parameters are altered systematically by varying throughput and screw speed. Based on this, a predictive model of a previous study [15] was extended to additional operating ranges at different specific feed loads. Furthermore, the prediction of residence time distributions was implemented to consider kinetic aspects that are particularly relevant when preparing pharmaceutical formulations via hot melt extrusion. Based on this novel modeling approach, design spaces were defined that predict the melt temperature and residence time depending on the screw speed and mass flow rate, as well as on the specific material parameters. Utilizing this new tool, it is possible to predefine process conditions for hot melt extrusion with a minimum number of experimental trials.

## Figures and Tables

**Table 2:** Results from the design of experiments: coefficients for factors (temperature (T), specific feed load (SFL) and used material (SOL, bBMA)) to the response variables (coefficient ± confidence interval (α = 0.05)) as well as the power of the model (coefficient of determination (R^2^), the coefficient of prediction (Q^2^) and the repeatability (RP)).

Parameter	$V_{dead}$ [mL]	$V_{mix}$ [mL]	CV [-]
T (β1)	-	-	0.044 ± 0.265
SFL (β2)	16.27 ± 4.28	-	−0.024 ± 0.016
SOL (β3)	-	−6.3 ± 2.86	0.013 ± 0.013
bBMA (β4)	-	4.7 ± 2.86	
R^2^	0.659	0.419	0.567
Q^2^	0.606	0.298	0.367
RP	0.887	0.837	0.807

**Table 3:** Model parameters of the temperature prediction. Linear regression models using slope (s) and intercept (i) in accordance with Equations (12) and (13). The coefficient of determination (R) is used as a performance parameter.

Substance	$s_{shear\;stress}$ [−]	$i_{shear\;stress}$ [−]	$R_{shear\;stress}$ [−]	$s_{extruder}$ [−]	$i_{extruder}$ [−]	$R_{extruder}$ [−]
PVPVA	6.77	158	0.936	0.417	0.114	0.949
SOL	5.35	254	0.913	0.214	−0.054	0.907
bBMA	6.54	157	0.981	0.011	−0.034	0.134
together	5.97	204	0.942	-	-	-

## Data Availability

The raw data supporting the conclusions of this article will be made available upon request.

## Appendix A

**Table A1:** Data of the extrusion experiments—design of experiments.

Polymer	n [1/min]	$\dot{m}$ [kg/h]	T [°C]	p [MPa]	k [1/s]	$t_{dead}$ [s]	σ [s]
PVPVA	60	3	171.0	1.50	0.0290	124.6	13.35
	74	3	171.7	1.49	0.0307	112.0	12.41
	120	3	175.3	1.34	0.0254	85.6	10.45
	120	6	179.7	1.91	0.0540	80.2	11.62
	148	6	180.0	1.71	0.0378	69.5	9.47
	148	6	180.7	1.78	0.0429	61.4	7.15
	148	6	180.3	1.76	0.0352	68.8	8.12
	239	6	186.3	1.44	0.0430	44.0	5.85
	239	12	189.7	1.73	0.0595	36.7	3.03
	296	12	192.3	1.82	0.0814	27.1	2.40
	478	12	198.3	1.45	0.0878	22.8	4.62
SOL	66	3	162.7	1.76	0.0348	114.7	15.37
	82	3	164.3	1.68	0.0287	106.0	13.46
	132	3	167.7	1.46	0.0340	91.4	14.05
	132	6	171.0	2.09	0.0603	59.8	7.54
	163	6	172.3	1.96	0.0544	53.9	6.74
	163	6	173.0	1.89	0.0457	52.7	4.84
	163	6	174.0	1.81	0.0549	56.3	7.57
	264	6	178.0	1.48	0.0801	48.0	8.11
	264	12	181.7	2.22	0.1278	30.5	4.53
	327	12	185.3	2.00	0.1324	28.7	4.94
	527	12	191.7	1.40	0.1983	27.9	6.02
bBMA	87	3	152.3	1.62	0.0242	115.2	13.03
	108	3	153.3	1.63	0.0245	111.4	12.37
	175	3	154.3	1.54	0.0247	91.1	11.09
	175	6	156.7	1.81	0.0456	56.1	6.33
	217	6	159.7	1.71	0.0437	51.9	6.61
	217	6	160.0	1.74	0.0417	50.3	6.99
	217	6	161.0	1.65	0.0402	48.5	6.73
	349	6	165.0	1.44	0.0407	44.7	6.81
	349	12	165.7	1.87	0.0673	25.4	3.22
	433	12	168.7	1.70	0.0988	23.7	3.09
	699	12	173.3	1.41	0.0764	17.3	3.56
